# An Association of Chitinase-3 Like-Protein-1 With Neuronal Deterioration in Multiple Sclerosis

**DOI:** 10.1177/17590914231198980

**Published:** 2023-12-07

**Authors:** Intakhar Ahmad, Stig Wergeland, Eystein Oveland, Lars Bø

**Affiliations:** 1 Department of Clinical Medicine, 1658 University of Bergen, Bergen, Norway; 2 Norwegian Multiple Sclerosis Competence Centre, Department of Neurology, 60498 Haukeland University Hospital, Bergen, Norway; 3 Norwegian MS-registry and biobank, Department of Neurology, Haukeland University Hospital, Bergen, Norway; 4 Neuro-SysMed, Haukeland University Hospital, Bergen, Norway; 5 Proteomics Unit at the University of Bergen (PROBE), Department of Biomedicine, 1658 University of Bergen, Bergen, Norway; 6 115347 Institute of Marine Research, IMR, Bergen, Norway

**Keywords:** chitinase-3-like protein 1 (CHI3L1), multiple sclerosis, cuprizone model, inflammation, demyelination, neurodegeneration

## Abstract

Elevated levels of Chitinase-3-like protein-1 (CHI3L1) in cerebrospinal fluid have previously been linked to inflammatory activity and disease progression in multiple sclerosis (MS) patients. This study aimed to investigate the presence of CHI3L1 in the brains of MS patients and in the cuprizone model in mice (CPZ), a model of toxic/metabolic demyelination and remyelination in different brain areas. In MS gray matter (GM), CHI3L1 was detected primarily in astrocytes and in a subset of pyramidal neurons. In neurons, CHI3L1 immunopositivity was associated with lipofuscin-like substance accumulation, a sign of cellular aging that can lead to cell death. The density of CHI3L1-positive neurons was found to be significantly higher in normal-appearing MS GM tissue compared to that of control subjects (*p*  =  .014). In MS white matter (WM), CHI3L1 was detected in astrocytes located within lesion areas, as well as in perivascular normal-appearing areas and in phagocytic cells from the initial phases of lesion development. In the CPZ model, the density of CHI3L1-positive cells was strongly associated with microglial activation in the WM and choroid plexus inflammation. Compared to controls, CHI3L1 immunopositivity in WM was increased from an early phase of CPZ exposure. In the GM, CHI3L1 immunopositivity increased later in the CPZ exposure phase, particularly in the deep GM region. These results indicate that CHI3L1 is associated with neuronal deterioration, pre-lesion pathology, along with inflammation in MS.

## Introduction

Chitinase-3-like protein-1 (CHI3L1), also known as YKL-40, is a glycoprotein primarily expressed in various tissues and cells, including macrophages, neutrophils, chondrocytes, and endothelial cells (Bonneh-Barkay et al., 2010; Recklies et al., 2002). This protein has garnered significant attention in the field of neuroscience due to its involvement in various physiological and pathological processes in the central nervous system (CNS) (Pinteac et al., 2021). CHI3L1 plays a role in multiple sclerosis (MS) pathogenesis, both clinically and experimentally. It has been implicated in functions such as inflammation, tissue injury, repair, and remodeling across different tissues (Lee et al., 2011; Zhao et al., 2020).

In the clinical context, CHI3L1 has emerged as an important biomarker in MS. Increased expression levels of CHI3L1 have been observed in the cerebrospinal fluid (CSF) of individuals with MS. It has been reported that elevated CSF levels of CHI3L1 are associated with an increased risk of conversion from clinically isolated syndrome (CIS-MS) to clinically definite MS (Roslind & Johansen, 2009). Furthermore, the extent of disability in patients with MS has been shown to be correlated with increased levels of CHI3L1 in CSF (Comabella et al., 2010). Pérez-Miralles et al. (2020) demonstrated that elevated CSF levels of CHI3L1 are predictive of further disability development in primary progressive MS (PPMS) patients. Gil-Perotin et al. (2019) suggested that combined measurement of CHI3L1 and neurofilament light chain protein (NFL) can help differentiate MS phenotypes and predict clinical progression. Additionally, Floro et al. (2022) conducted a meta-analysis indicating that relative increases in CSF levels of CHI3L1 may distinguish PPMS from relapsing-remitting MS (RRMS) and secondary progressive MS. In a recent study, CHI3L1 has been suggested as a potentially useful biomarker for treatment decision-making in MS patients (Lucchini et al., 2023). In the study by Lucchini et al., multiple neuroinflammatory markers in the CSF were assessed in a large cohort of RRMS patients. The findings demonstrated that the group with high concentrations of both CXCL13 and CHI3L1 had a significantly higher risk of relapse, increased MRI activity, and evidence of disease activity during follow-up. Hence, the authors concluded that CSF levels of CXCL13 and CHI3L1 serve as strong prognostic markers in RRMS patients.

Experimental studies have provided insights into the functioning of CHI3L1 in the CNS. *In vitro* experiments have demonstrated that CHI3L1 exhibits a neuron-specific cytotoxic effect, leading to neurite retraction and reduced neuronal survival (Huang et al., 2014; Matute-Blanch et al., 2020). Furthermore, Jiang et al. (2019) reported that CHI3L1 promotes the proliferation of oligodendrocyte precursor cells.

The cellular origin of CHI3L1 within the CNS remains an area of interest. Immunohistochemical studies have identified CHI3L1 immunopositivity in astrocytes and macrophages/microglia in MS brain tissue, particularly in active and chronic active white matter (WM) lesions (Cubas-Núñez et al., 2021; Hinsinger et al., 2015). However, a systematic assessment of CHI3L1 distribution in gray matter lesions (GMLs) and normal-appearing gray matter (NAGM) in MS has not been conducted to date.

Studies on the role of CHI3L1 in demyelination have been reported in the experimental autoimmune encephalomyelitis (EAE) mouse model. Mouse breast regression protein 39 (BRP-39) is homologous to the human CHI3L1 protein (Cantó et al., 2015). In one study, BRP-39 knockout female mice with EAE showed increased clinical scores and immunopathological markers of CNS inflammation compared to controls (Bonneh-Barkay et al., 2012). However, another study using a combination of male and female mice and a different dose of antigen peptide found no difference in the clinical course of EAE between wild-type and BRP-39 knockout mice of the same mouse strain (Cantó et al., 2015). Although the EAE model provides insights into immune-mediated mechanisms and therapeutic interventions for MS, it primarily mimics the relapsing-remitting form of MS and does not fully capture the progressive aspects and long-term neurodegeneration seen in the disease (Giovannoni et al., 2017). On the other hand, the cuprizone (CPZ) model involves the administration of cuprizone, a copper chelator that induces demyelination in a toxicological manner (Skripuletz et al., 2011). The CPZ model exhibits features resembling the progressive forms of MS, making it suitable for studying progressive disease processes (Matsushima & Morell, 2001). To our knowledge, there are no studies published on the involvement of CHI3L1 in the pathogenesis of demyelination/neurodegeneration in the CPZ model.

Therefore, the purpose of our study is to investigate the distribution of CHI3L1-expressing cells in both the MS brain and a mouse model of toxin-induced de- and remyelination known as the CPZ model. By examining CHI3L1 expression patterns in MS lesions, including gray matter (GM) lesions, we aim to further elucidate the role of CHI3L1 in MS pathogenesis. Additionally, this study aims to contribute to the current knowledge by providing a comprehensive assessment of CHI3L1 distribution in the CNS. The novelty of our study lies in the systematic evaluation of CHI3L1 immunopositivity in GMLs and NAGM in MS by expanding the assessment beyond WM lesions.

## Materials and Methods

### Human Brain Tissue Samples

In this study, we have included autopsy material from nine MS cases, along with five controls with no known CNS disease and no histopathological signs of infection, inflammation, or neurodegeneration. Demographic and clinical data were collected retrospectively (Table 1). The brain tissue samples were obtained from The Norwegian MS Registry and Biobank in the Department of Pathology, Haukeland University Hospital, Bergen, Norway. The study was approved by the institutional review board for medical research ethics of Western Norway (permit No: 2013-560). Consent to use tissue for research was taken in writing, either by the patient or after death by the nearest living relative. The medical records tagged with personal information were anonymized before those were used for research.

**Table 1.** Clinical and Demographic Description of MS Brain Autopsies and Control Cases.

MS	MS phenotype	Gender	Age at autopsy (years)	Disease duration (years)	Cause of death
1	Progressive	Male	43	7	Bronchopneumonia
2	Progressive	Male	34	13	Bronchopneumonia
3	Progressive	Male	43	7	Bronchopneumonia
4	Progressive	Male	46	12	Suicide
5	Relapsing-remitting	Female	52	20	Bronchopneumonia
6	Progressive	Female	45	8	Acute pyelonephritis with sepsis
7	Progressive	Male	52	8	Acute pyelonephritis
8	Progressive	Male	50	20	Bronchopneumonia
9	Progressive	Female	45	8	Acute pyelonephritis with sepsis
Control					
1		Female	37		Epilepsy^a^
2		Male	53		Diabetes mellitus
3		Female	47		Heart disease (HSCRT)
4		Male	40		Endocarditis
5		Female	30		Lymphangioleiomyomatosis (LAM)

*Note*. MS patients may have exhibited higher EDSS values.

The available information on the disease course of the MS cases included in the study is limited, as many patients with progressive disease of long duration are not followed regularly at the department of neurology but are being treated by the nursing home physician. Infection as a cause of death indicates that the patients were likely to be severely disabled. MS  =  multiple sclerosis; CNS = central nervous system.

^a^ The epilepsy patient had no prior history of CNS disease, indicating that the seizure occurred without any identifiable pre-existing structural or functional abnormalities in the brain, classifying it as cryptogenic epilepsy. Furthermore, in terms of CHI3L1  +  cell distribution, the epilepsy sample did not exhibit a significant difference compared to the distribution observed in the four other control samples.

Particular attention was given to select brain samples from MS patients who were not too old to prevent the confounding effects of age-related CNS pathology. The median age of MS patients was 45 (min 34, max 52), while that of controls was 40 (min 30, max 53). However, the MS patients had a minimum disease duration of 7 years, which is sufficient to investigate disease progression.

### Human Brain Histopathology

For human brain histopathology, sections (5–6 µm) from paraffin-embedded blocks were immunohistochemically stained for target proteins. For immunohistochemistry, the sections were dewaxed with xylene and then rehydrated through gradients of ethanol and into water. For antigen retrieval, sections were incubated in the Diva Decloaker antigen retrieval solution (DV2004LX, Biocare Medical, CA, USA) at pH 6.2; 120°C; at 15 psi for 15 min. Primary antibodies used for immunohistochemistry were myelin proteolipid protein, PLP (1:1000, Rabbit monoclonal, Abcam, RRID: AB_2915963; overnight at 4°C), HLA-DR (1:20, mouse monoclonal, DAKO, RRID:AB_2262753; 2h at RT), and CHI3L1 (1:200, Rabbit polyclonal, Abcam, RRID: AB_2891040; 24h at 4°C). The sections were blocked with a blocking solution (EnVision FLEX Peroxidase-Blocking Reagent, Dako, Glostrup) and visualized with EnVision  +  System (Dako, Glostrup) following the manufacturer's guideline (EnVision Systems: EnVision  +  Dual Link, Single Reagents; HRP. Rabbit/Mouse). The tissue sections were finally counterstained with hematoxylin before mounting permanently in dibutylphthalate polystyrene xylene. The omission of the primary antibody acted as a negative control. The Sudan Black B (Sigma-Aldrich®) lipid staining, widely used to react against lipofuscin aggregates in cells, was applied to tissue sections from controls and MS patients containing cerebral cortex (Evangelou & Gorgoulis, 2017). Multiplex staining was performed using both immuno-enzymatic and immunofluorescence techniques, depending on the visual distinction required. Using the same primary antibodies, immuno-enzymatic double labeling was carried out with the EnVision  +  Dual Link System-HRP (Agilent Technologies Inc.) according to the manufacturer's instructions. In the immunofluorescence procedure, the sections were first deparaffinized and then blocked using PBS and 5% BSA before being stained in a humidified chamber at RT. The primary antibodies used were identical to single stainings. To label the primary antibodies, the VectaFluor™ Duet Immunofluorescence Double Labeling Kit was utilized, and DAPI (Invitrogen™) was applied for nuclear counterstaining. In our study, we identified neurons in the cortex based on their well-known distinct morphological features, which have also been highlighted by García-Cabezas et al. (2016).

### Lesion Identification, Categorization, and Quantification of CHI3L1  +  Cell Density in Human Brain

Gray and WM lesions were analyzed separately (*n*  =  57). GMLs were initially categorized according to the Bø-Trapp classification system (Bø et al., 2003a, 2003b). Since mixed WM-GM lesions (type I) have immune cell infiltrates (Bø et al., 2003b), GM lesions have been grouped into mixed WM-GM lesions (*n*  =  6) and purely intracortical lesions, types II, III, and IV (*n*  =  19). For WM lesions, lesions were classified according to inflammatory activity (Bö et al., 1994). In the WM part, HLA-DR staining was the basis for categorizing lesions (*n*  =  32) as either active, chronic active, or inactive, and corresponding PLP staining was used to identify MS lesion areas (Ahmad et al., 2021; Oveland et al., 2021). Early active, active, and chronic active WM lesions were put in the active lesion group (*n*  =  15). Immunopositivity was counted as a cumulative value of positive cell profiles in three random visual fields of 0.0625 mm^2^ each in regions of interest by light microscopy (Zeiss Axio Imager A2, Wetzlar, Germany) using an ocular morphometric grid. Since cortical neurons in the controls were very weakly immunopositive for CHI3L1, careful thresholding was applied compared with neighboring neurons.

### Cuprizone Experiment

For the CPZ experiment, 48 female C57BL/6 mice were obtained from Taconic Biosciences (Tornbjerg, Denmark) at the age of 7 weeks. It has been reported that there is no significant difference in the demyelination severity induced by CPZ in male and female C57BL/6 mice (Vega-Riquer et al., 2019). During the acclimatization and experimental periods, they were housed in six per cage in Macrolon IVC-II cages (Scanbur, Karlslunde, Denmark). After 1 week of adaptation to the laboratory environment, 8-week-old mice were randomized to eight groups of six mice and exposed to 0.2% (w/w) CPZ (bis-cyclohexanone-oxaldihydrazone, Sigma-Aldrich, St. Louis, MO) in mouse diet for up to 6 weeks. Food and tap water were provided ad libitum throughout the experimental period. Mice were sacrificed weekly (six per week) during CPZ exposure, and the remaining group (*n*  =  6) was sacrificed 2 weeks after ending CPZ exposure. Control mice (*n*  =  6) were sacrificed at the onset of the experiment. To minimize suffering and distress for the animals, their cages were provided with bedding, nesting material, shelters, and chewing implements. Pain relief and, thus a need for analgesics was not expected in this study. In one experiment group, one mouse out of a group of six died after 3 weeks of CPZ exposure, and that mouse was excluded from the analysis. The experiment was conducted following the recommendations of the Federation of European Laboratory Animal Science Associations and the ARRIVE guidelines (du Sert et al., 2020). The Norwegian Animal Research Authority approved the experimental protocol.

### Mouse Brain Histopathology

Mice were euthanized by CO2 asphyxiation, and collected brains were post-fixed in 4% neutral-buffered formalin for 7 days, then embedded in paraffin. All analyses were performed on 5 µm coronal sections from the bregma + 1 mm. The CPZ sections were incubated with primary antibodies against myelin PLP (1:1000, Rabbit monoclonal, Abcam, RRID: AB_2915963; overnight at 4°C), MAC3 (1:200, Rat monoclonal, BD Biosciences, RRID: AB_394780; 24 h at 4°C), CHI3L1(Brp39) (1:200, Rabbit polyclonal, Abcam, RRID: AB_2891040; 24 h at 4°C). The sections were blocked with peroxidase-blocking solution (Dako, Glostrup) and visualized with the EnVision  +  System (Dako, Glostrup, DK). The tissue sections were counterstained with hematoxylin. The omission of the primary antibody was used as a negative control for the secondary antibody following the same immunohistochemical methods used for human samples.

### Quantification of CHI3L1 + , MAC3  +  Cell Density, and Myelin Content in CPZ Mouse Brain

In the CPZ model, the density of CHI3L1 and MAC3 immunopositive cells in GM (cerebral cortex), in WM (corpus callosum), and in a mixed tissue area (deep GM) were examined by light microscopy (Zeiss Axio Imager A2, Wetzlar, Germany) using an ocular morphometric grid on three random areas. The ocular morphometric grid covered an area of 0.0625 mm^2^. For the cortex, systematically randomized areas in the primary somatosensory cortex within layers 2–5 were quantified. Cell densities were the nearest whole number of the average of three randomly selected areas in the cortex and the deep GM. For the corpus callosum, a single count from each of the two lateral and the medial part. Counts were averaged to a single integer value.

## Statistical Analysis

Due to the non-normal distribution pattern of the data, statistical analysis was performed using the Wilcoxon sign rank test to evaluate pairwise differences between different lesion groups within the same tissue samples in human specimens. The Kruskal–Wallis test was utilized to determine the overall difference between the control and MS tissue samples. For CPZ samples, the temporal changes in CHI3L1  +  cell density in different brain tissue regions during demyelination were analyzed using one-way analysis of variance with Tukey's HSD test. An independent-sample *t*-test was applied to determine the statistical significance of the differences between the sample group and the control group. The Pearson Correlation Coefficient (r) was calculated to assess the relationship between cell densities and various immunostainings across the entire experimental period in different tissue regions. All analyses were performed using GraphPad Prism version 9.0 (GraphPad Software Inc., San Diego, CA). Results were considered significant at *p* < .05.

## Results

### CHI3L1 Expression Pattern in Human Grey Matter Tissue

**Figure 1.**
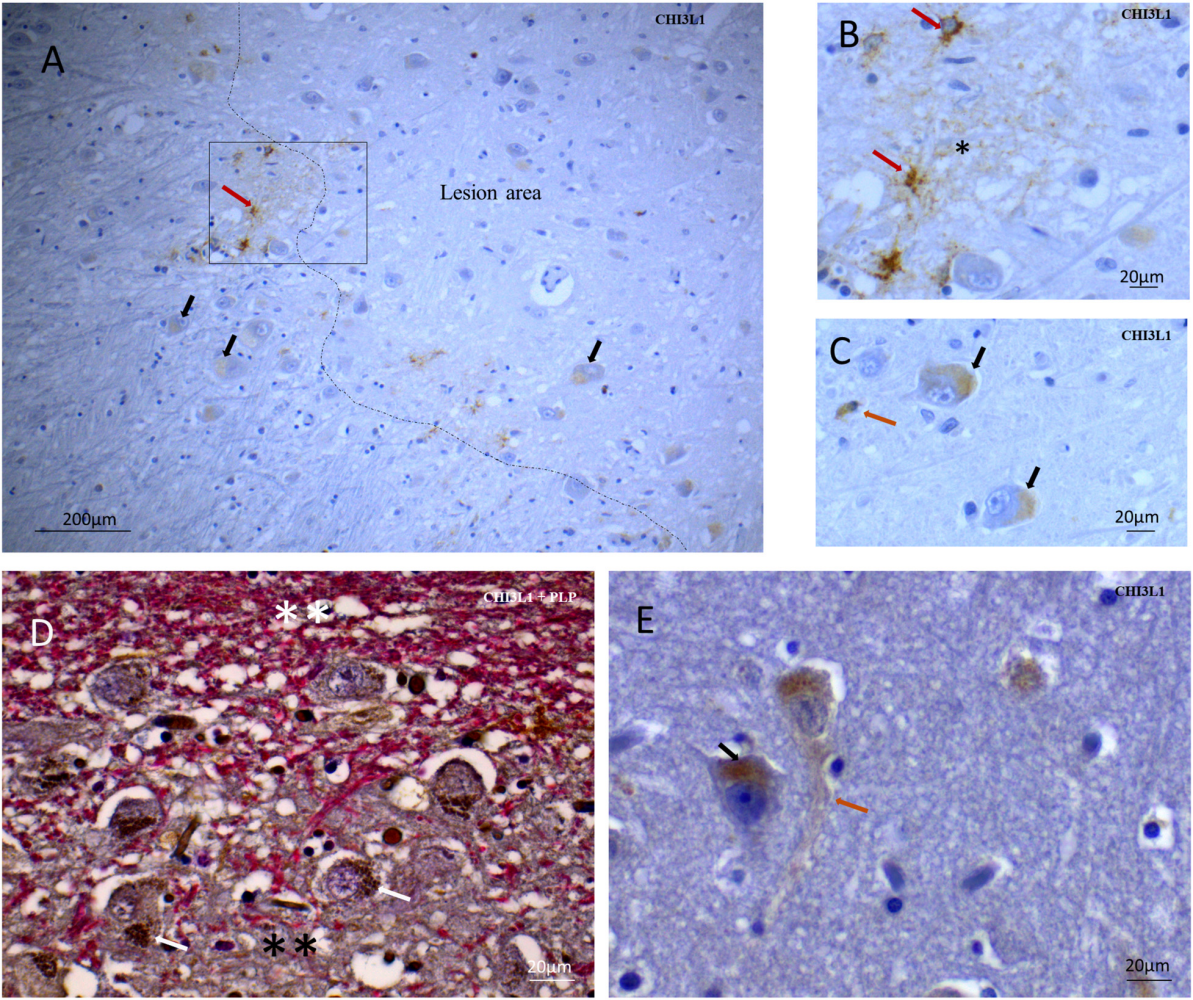
Pattern of CHI3L1 positivity in MS GM tissue part. (A) GM area stained with CHI3L1 antibody. Immunopositivity is observed in the lesion area and near the lesion area with shadows of myelin fibers (marked with a dashed line) and strongly CHI3L1  +  reactive astrocytes (red arrow). Cytoplasmic material in neurons is faintly positive for CHI3L1(black arrow). (B) The rectangular box in 1A is presented in higher magnification. There are strongly CHI3L1  +  reactive astrocytes (red arrow), and the single asterisk represents a positive extracellular matrix for CHI3L1. (C) CHI3L1-positive cytoplasm in neurons (black arrow) and a cell with the morphology of a resting astrocyte (orange arrow). (D) Double stained, Myelin protein-PLP (red) and CHI3L1(brown), CHI3L1  +  aggregates in neuronal soma (white arrow) on a GM lesion border (black double asterisk represents the demyelinated area and white double asterisk represents normal-appearing myelination) (E) CHI3L1  +  neuronal soma (black arrow) and axon (orange arrow) in a normal-appearing cortex area. CHI3L1 = Chitinase-3-like protein-1; PLP = proteolipid protein.

In MS brains, CHI3L1 immunopositivity was not restricted to the GM lesion areas; diffuse immunopositivity was observed throughout the cerebral cortex (Figure 1). The frequency and extent of CHI3L1 immunopositivity were increased in and around GM lesions (Figure 1(A) and 1(D)). Strong CHI3L1 immunopositivity was frequently detected in cells with reactive astrocyte morphology and in astrocyte-like cell processes in the extracellular matrix areas around the clusters of strongly CHI3L1-positive reactive astrocyte looking cells (Figure 1(A) and (B)). It is already established through hybridization studies that, *in vivo*, the transcription of CHI3L1 is primarily connected with astrocytes (Bonneh-Barkay et al., 2010). Additionally, we found that a minor subset of pyramidal neurons and a small minority of cells having the morphology of non-activated astrocytes were less intensely immunopositive for CHI3L1 (Figure 1(A) and (C)). The extracellular matrix adjacent to neurons or non-activated astrocytes was not immunopositive for CHI3L1 (Figure 1(A), (C), and (E)). In neurons, in and around GM lesions, granular CHI3L1  +  vesicles were observed in the cell body cytoplasm around the nucleus (Figure 1(D) and (E)). Rarely, axons were weakly immunopositive for CHI3L1 (Figure 1(E)).

**Figure 2.**
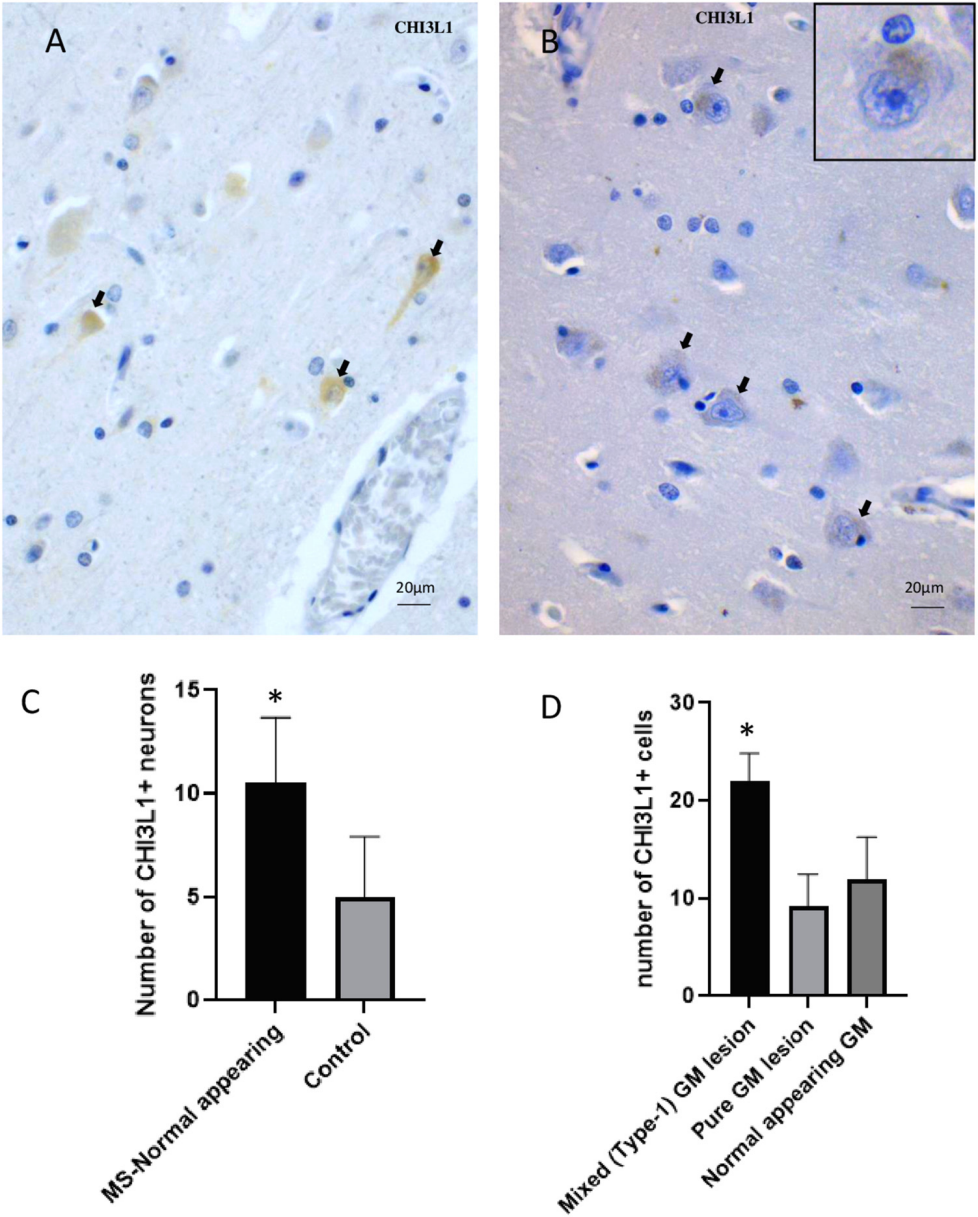
Differential distribution of CHI3L1  +  cells in non-lesional MS GM and control GM. (A) A NAGM area immunostained for CHI3L1, some positive neurons (back arrow). (B) A control GM area stained with CHI3L1, some faintly positive neuronal soma (black arrow); inset highlights the pattern of positivity in higher magnification. (C) Distribution of CHI3L1-positive neurons in NAGM parts in MS and in controls (mean, SEM). (D) Density of CHI3L1-positive cells in different types of GMLs and normal-appearing tissue parts (mean, SEM). The Kruskal–Wallis test assessed overall differences between control and MS tissue samples. The Wilcoxon sign rank test compared pairwise differences within lesion groups. CHI3L1 = Chitinase-3-like protein-1; NAGM = normal-appearing gray matter; GM = gray matter; MS = multiple sclerosis. *Significantly different from other group/groups at *p* < .05.

Normal-appearing MS cortex and control cortex also contained CHI3L1-immunopositive neurons (Figure 2(A) and (B)). Though, the control cortex was very weakly positive for neurons (Figure 2(B)). The density of CHI3L1-immunopositive neurons was significantly higher (*p*  =  .014) in normal-appearing MS tissue when compared to controls (Figure 2(C)). The density of CHI3L1-immunopositive cells was significantly higher in the GM part of mixed GM/WM lesions (type I) than in pure GM lesions and normal-appearing cortex (Figure 2(D)).

### Features of CHI3L1  +  Cells

**Figure 3.**
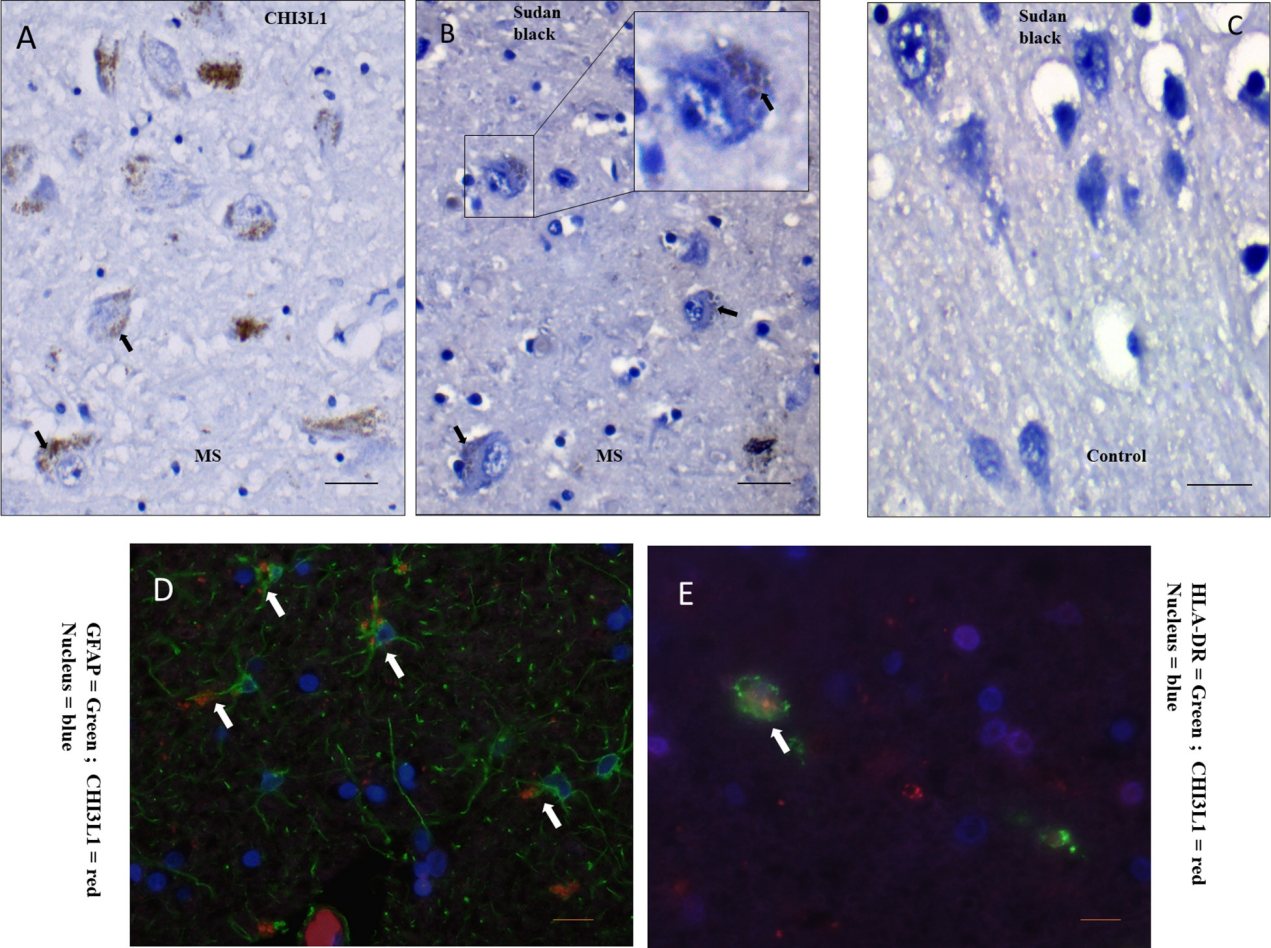
Cells having CHI3L1 positivity and their features. (A) In a GM (cortex), near-lesion area with CHI3L1 staining, large pyramidal neurons contain granules positive for CHI3L1 (black arrow). (B) In the matching area on the same tissue block, Sudan Black-positive neurons (black arrow) that confirm the presence of lipofuscin vesicles in cellular location of CHI3L1 positivity. (C) Sudan Black staining of control GM shows negligible amount of positivity. (D) Immunofluorescent staining to observe colocalization of the cell type marker, GFAP for astrocytes (green) and chitinase (red). (E) Immunofluorescent staining to observe colocalization of the cell type marker, HLA-DR for antigen-presenting microglia/macrophages (green) and chitinase (red). Scale bar  =  20 μm. CHI3L1 = Chitinase-3-like protein-1; GM = gray matter.

The CHI3L1  +  granular structures have an association in pyramidal neurons having lipid-containing residues (lipofuscins), as identified by relating CHI3L1 and Sudan black staining pattern of the same region of the same tissue blocks with the help of morphological details at subcellular level (Figure 3(A) and (B)). Negligible or absent Sudan black staining was evident in the control GM samples (Figure 3(C)). The co-labeling of CHI3L1 and lipofuscin was not feasible due to incompatible methodologies. It is noteworthy that only a subset of neuronal cells had lipofuscin vesicles stained by Sudan black, and no Sudan black-positive glial or infiltrating cells were observed in the cortex.

Colocalization of CHI3L1 was detected on GFAP  +  cells (astrocytes), primarily in cellular processes (Figure 3(D)). HLA-DR  +  cells were also positive for CHI3L1 (Figure 3(E)). It was evident that CHI3L1 positivity was predominant in reactive astrocytes; however, not all reactive astrocytes were CHI3L1 positive (Figure 3(D)).

### CHI3L1 Expression Pattern in Human White Matter Tissue

**Figure 4.**
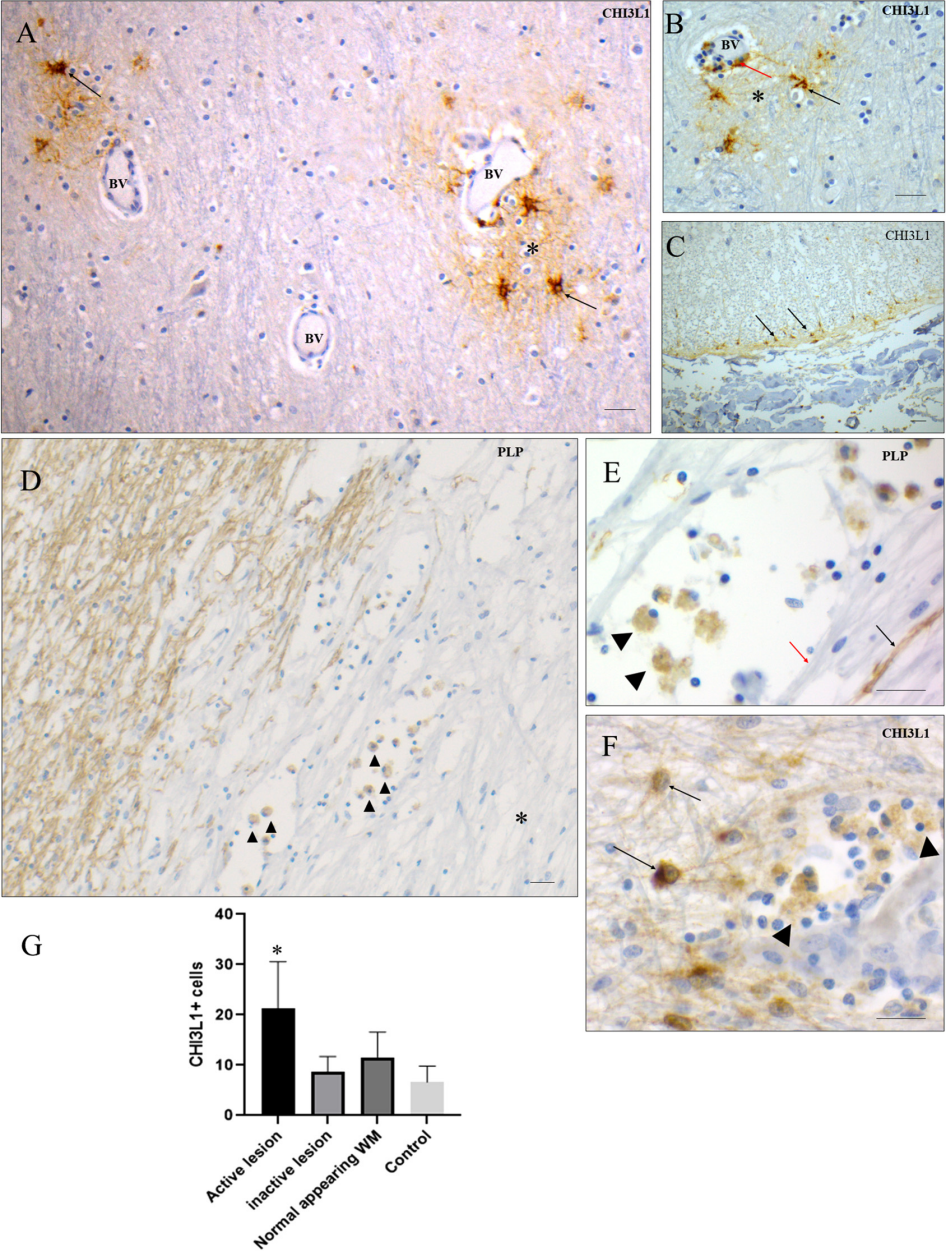
CHI3L1  +  cell density and distribution pattern in different WM tissue parts. (A) In a WM normal-appearing area, a population of CHI3L1  +  astrocytes (black arrow) around BVs, CHI3L1-positive extracellular matrix (the single asterisk mark). (B) A closer view of the BV area, where there are CHI3L1  +  astrocytes (black arrow), positive extracellular matrix (* mark), and positive cells in the perivascular area (red arrow). (C) Cells of astrocyte morphology in the subpial layer of the spinal cord are positive for CHI3L1 (black arrow). (D) PLP staining shows active lesion areas where positive phagocytic cells contain myelin remnants (black arrowhead) which is a feature of an early stage of lesion formation. (E) PLP staining shows teaming of phagocytic cells (arrowhead) having a mixture of myelinated (black arrow) and demyelinated (red arrow) fibers of axons. (F) Reactive astrocytes (black arrow) expressing CHI3L1 were found alongside foamy macrophages (arrowhead). (G) Density of CHI3L1  +  cells in different types of WM lesions and normal-appearing tissue parts and WM of controls (mean, SEM). Given the non-normal data distribution, the Wilcoxon sign rank test compared pairwise differences within lesion groups in human tissue samples. Scale bar  =  20 μm. CHI3L1 = Chitinase-3-like protein-1; BVs = blood vessels; PLP = proteolipid protein; WM = white matter.

In MS normal-appearing white matter (NAWM), cellular and extracellular CHI3L1 immunopositivity was frequently observed both in the lining and immediately outside of the perivascular space (Figure 4(A) and (B)). Not all blood vessels (BVs) were positive for CHI3L1  +  cells, however, most of the CHI3L1  +  cells in the vicinity of BVs had an activated astrocyte morphology (Figure 4(A)). Like in GM areas, extracellular matrix areas around clusters of strongly positive astrocytes were immunopositive for CHI3L1, morphologically consistent with astrocyte processes (Figure 4(B)). In MS spinal cord, the density of CHI3L1-positive astrocytes was increased in subpial WM areas (Figure 4(C)). In early-stage WM MS lesions, cells with the morphology of foamy macrophages (containing abundant intracellular myelin remnants, identified by PLP positivity) were immunopositive for CHI3L1 (Figure 4(D) and (E)). Foamy macrophages were accompanied by CHI3L1 astrocytes (Figure 4(F)). In control brains, only very few cells were immunopositive for CHI3L1 compared to MS-WM (Supplementary Figure 1). Since the borders of active/chronic active WM lesions were densely infiltrated by CHI3L1  +  phagocytic macrophages, the density of CHI3L1  +  cells were significantly higher in active/chronic active WM lesions compared to inactive lesions, NAWM areas, and control WM (Figure 4(G)).

### Temporal Variation and Distribution of CHI3L1 Expression During and After Cuprizone Exposure in Mice

**Figure 5.**
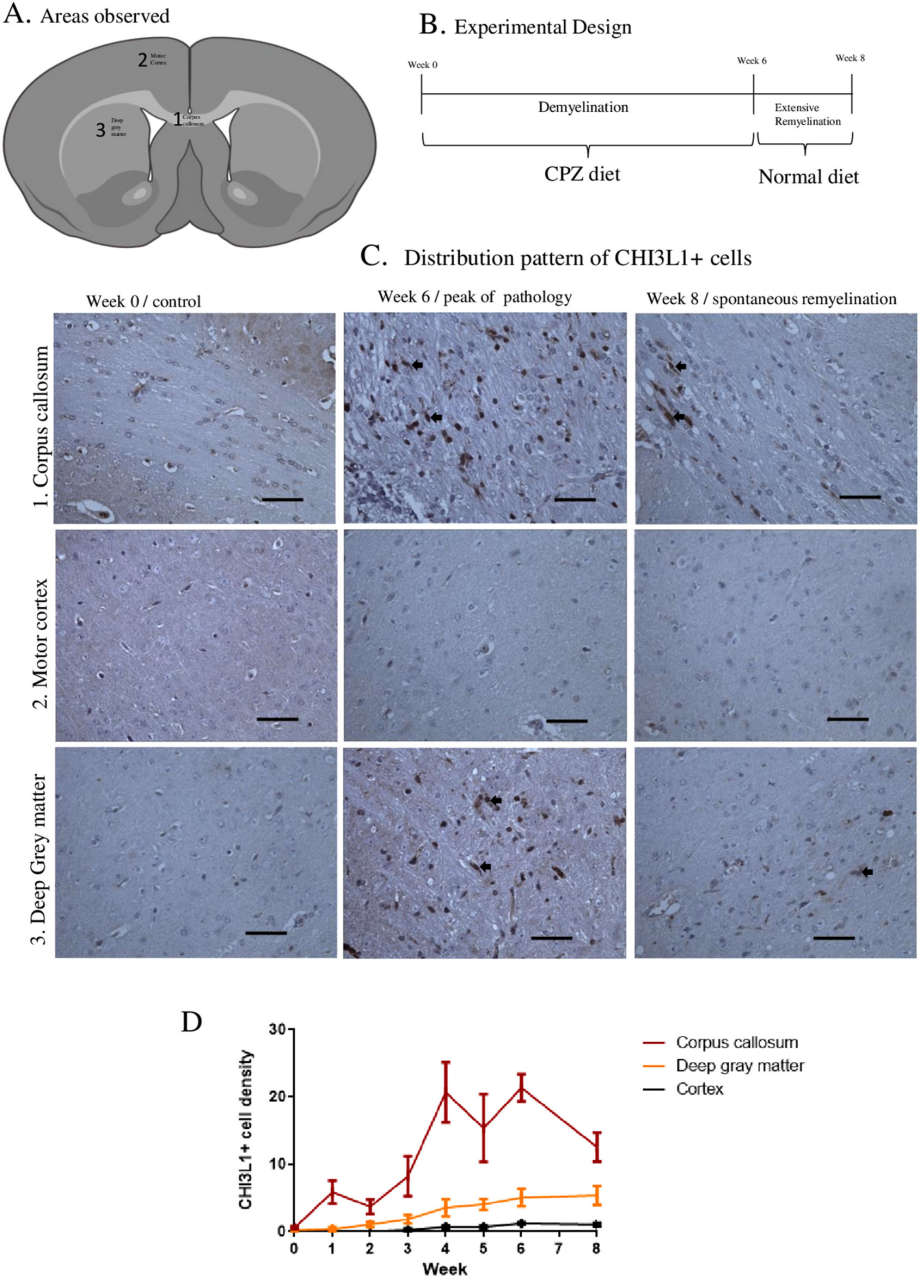
Spatio-temporal CHI3L1  +  cell densities in the CPZ experiment. (A) Areas of the mouse brain taken into analysis, the corpus callosum, motor cortex, and deep GM (mixed tissue type). (B) Schematics of the CPZ experiment, the beginning (week 0), disease peak (week 6) and 2 weeks after CPZ exposure (week 8). (C) Distribution pattern of CHI3L1  +  cells in the areas of interest at different time points, the corpus callosum represents WM and the motor cortex signifies purely GM tissue, while deep GM demonstrates a mixture of WM and GM tissue. (D) Graphs show the dynamics of CHI3L1  +  cells throughout the CPZ experimental period (mean, SEM). Scale bar  =  20 μm. CHI3L1 = Chitinase-3-like protein-1; CPZ = cuprizone; WM = white matter; GM = gray matter.

Study areas are marked in the Figure 5(A) and the study design is schematically explained in the Figure 5(B). The majority of CHI3L1-immunopositive cells in the WM of the CPZ mice had microglial morphology. However, in the WM of control mice, almost no CHI3L1 immunopositivity was present, except for some immunopositivity within BVs (Figure 5(C)). The increase in CHI3L1 immunopositivity was more pronounced and occurred earlier in the corpus callosum than in deep GM or cerebral cortex, yet a gradual increase of CHI3L1 immunopositivity was also observed in the deep GM during the entire experimental period (Figure 5(C) and (D)). White and GM parts showed profound differences in the pattern of CHI3L1 positivity. In the cortex, the increase of immunopositivity was slower and less pronounced, and went back almost to pre-exposure levels after ending the CPZ exposure (Figure 5(C) and (D)). The density of CHI3L1  + cells declined sharply in the corpus callosum (*p*  =  .002). Unlike in the cortex, the WM (corpus callosum) decline did not reach the pre-exposure level.

**Figure 6.**
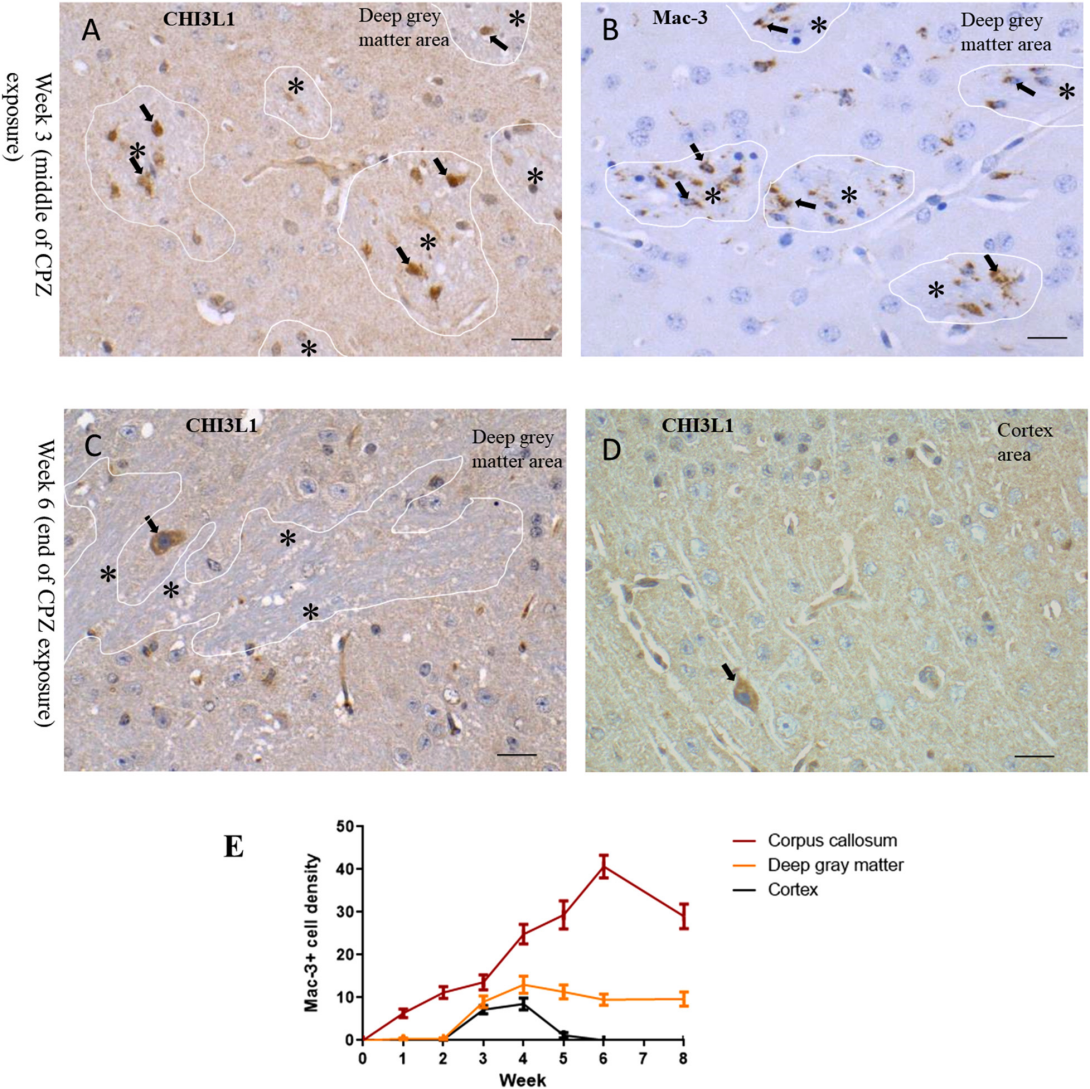
Distribution of CHI3L1  +  cells in relation to inflammatory marker Mac-3  +  cells in the CPZ experiment. (A) In the deep GM area (mixed white and GM) at week 3, CHI3L1 +  cells (black arrow) appeared only in the WM patches (marked with a white line and a single asterisk). (B) In the deep GM area at week 3, Mac3  +  cells (black arrow) showing microglia infiltration in WM patches (marked with a white line and a single asterisk). (C) In the deep GM area at week 6, CHI3L1  +  neurons (black arrow) appeared in the GM part, WM area is marked with a white line, and single asterisks. (D) At week 6, CHI3L1-positive neurons appeared sporadically in the deeper layer of the cortex (a positive neuron is marked with a black arrow). (E) Graphs show the dynamics of Mac-3  +  cells throughout the CPZ experimental period (mean, SEM). Scale bar  =  20 μm. CHI3L1 = Chitinase-3-like protein-1; WM = white matter; GM = gray matter; CPZ = cuprizone.

The density of Mac-3  +  cells in the corpus callosum increased during CPZ exposure and waned quickly after completing the exposure, which follows the pattern of CHI3L1 distribution. In deep GM, CHI3L1  +  cells appeared earlier in WM tracts, where microglia activation was observed (Figure 6(A) and (B)). On the contrary, CHI3L1  +  neurons were detected at a later stage of CPZ exposure (Figure 6(C)), which follows the pattern in the cortex area (Figure 6(D)). The increase in density of Mac-3  +  cells during CPZ exposure was less pronounced in the cortex (Figure 6(E)). Combination of white and GM tissue parts made the increase in Mac-3 positivity moderate in the deep GM, and Mac-3 immunopositivity was mostly confined to WM patches in the deep GM (Figure 6(B) and (E)). The density of CHI3L1  +  cells and Mac-3  +  cells was significantly correlated in the corpus callosum of the CPZ mice (*r*  =  .76, *p* < .05). CHI3L1 immunopositivity also showed a strong association with choroid plexus inflammation identified by Mac-3 immunopositivity (Supplementary Figure 2).

## Discussion

This study investigates the presence of CHI3L1 in the brains of MS (mostly PMS) patients and in the CPZ model in mice to characterize further the role of CHI3L1 in the pathogenesis of MS.

The findings are consistent with prior results indicating that the molecule plays a role in modulating the inflammatory response within the CNS. Furthermore, this study also discovered increased CHI3L1 immunopositivity in the cortex of individuals with MS.

While the role of CHI3L1 in the pathogenesis of MS is unknown, most studies on CSF samples from MS patients indicate that CHI3L1 may be a marker of CNS inflammation (Burman et al., 2016). The expression and/or secretion of CHI3L1 may be neurotoxic, as CHI3L1 concentrations similar to those measured in the CSF from active MS patients were mildly neurotoxic to primary cultured neurons in vitro (Matute-Blanch et al., 2020). The CSF level of CHI3L1 was correlated to cognitive impairment in the early stages of MS (Quintana et al., 2018). Clinically isolated syndrome (CIS) patients with high CSF levels of CHI3L1 have been reported to have a four times higher risk for the development of neurological disability, compared to CIS subjects with low CSF CHI3L1 levels (Canto et al., 2015). The observations above indicate an association of CHI3L1 with neuronal degeneration and/or decreased neuronal function in MS. This is supported by data from other neurodegenerative diseases. Elevated CHI3L1 level in CSF is a biomarker for Alzheimer's Disease development (Wennström et al., 2015). CHI3L1 expression levels are significantly upregulated in the motor cortex of patients with motor neuron disease in sporadic amyotrophic lateral sclerosis (Sanfilippo et al., 2017).

In this study, in GM, extensive CHI3L1 immunopositivity was observed in astrocytes in lesion- and near-lesion areas, and in granular cytoplasmic structures in pyramidal neurons (Figure 1). This is a similar pattern to what has recently been reported in Alzheimer's disease cortex (Hok-A-Hin et al., 2022). Although very faintly CHI3L1  +  neurons were detected in control brains, they were significantly more frequent in the MS brains, in the lesions, in near-lesion- and normal-appearing areas, compared to the control brains (Figure 2(C)).

In neurons, CHI3L1 immunopositivity was mainly confined in the subcellular areas that exhibit positivity for lipid vesicles (lipofuscin) (Figure 3(A) and (B)). Lipofuscin accumulation in the CNS is associated with ageing (Gray & Woulfe, 2005), interpreted to be a product of defective cellular metabolism, including mitochondrial dysfunction, incomplete mitophagy, altered proteostasis, protein misfolding and aggregation, and endolysosomal dysfunction (Moreno-García et al., 2018). The macroautophagy-lysosomal pathway is essential for maintaining protein and energy homeostasis (Lamark & Johansen, 2012). Dysfunctions of the lysosomal system have been implicated in neurodegenerative diseases with protein aggregation and mitochondrial dysfunction. The asparaginyl endopeptidase legumain is upregulated in neurons in Alzheimer's disease and in posttraumatic neurodegeneration. This molecule is considered as a biomarker and a potential therapeutic target for Alzheimer's disease (Song, 2022). Legumain is also activated in lysosomes and is also present in aggregates in the frontal cortex neurons of the MS brain (Oveland et al., 2021). Lipofuscin is a cytoplasmic accumulation of lipid and protein debris (Gray & Woulfe, 2005) . Endocytosis and endosomal sorting can bring extracellular macromolecules to lysosomes (Ferguson, 2019). Chitinase-like proteins can bind extracellular matrix components (Kognole & Payne, 2017), and membrane-associated proteins can be endocytosed within the neuron by several possible routes (Henaff & Salinas, 2010; Parton & Dotti, 1993). However, this study did not explore the source or the entry mechanism of CHI3L1 into neuronal cells.

In early active WM lesions, foamy phagocytic cells (macrophages) were positive for CHI3L1 (Figure 4(D) and (E)). CHI3L1 immunopositivity in macrophages was tightly associated with inflammation, as the inflammatory regions of active/chronic active WM lesions and the mixed GM/WM lesions had the highest density of CHI3L1  +  cells (Figures 2(D) and 4(G)).

In perivascular areas of normal-appearing WM in MS, astrocytes in perivascular areas were frequently immunopositive for CHI3L1 (Figure 4(A) and (B)). CHI3L1 immunopositivity may thus be associated with blood–brain barrier (BBB) integrity in the early stages of WM lesion development, as plasma protein leakage is a WM phenomenon, whereas plasma protein leakage is frequently absent in GMLs (Van Horssen et al., 2007).

In the CPZ model, CHI3L1 immunopositivity was closely related to the inflammatory response and mostly detected in cells with microglial morphology (Figure 5(C)). During the CPZ exposure phase, the spontaneous remyelination process starts around week 3 (Ahmad et al., 2021). Interestingly, the increase of CHI3L1 immunopositivity started to rise from that time point, even in GM parts (Figure 5(D)). This indicates that CHI3L1 might be associated with remyelination processes, as inflammatory cells participate in the early signaling of remyelination events (Skihar et al., 2009). The correlation of CHI3L1 with remyelination is supported by an observation indicating that molecules of the CHI3L1 family can induce oligodendrogenesis in the EAE model (Starossom et al., 2019).

CHI3L1  +  microglial cells appeared early in the WM fiber tracts embedded in the deep GM during the CPZ exposure period. This was followed by the appearance of CHI3L1  +  neurons in deep GM (Figure 6(A)–(C)). In the late stage of CPZ exposure, a few cortical neurons were also found immunopositive (Figure 6(D)). These data support an association of CHI3L1 neuronal immunopositivity with inflammation-related insults in neuronal cells.

The present study has limitations. Most autopsy MS samples were from the progressive MS phenotype, and the observed pattern of CHI3L1 immunopositivity needs to be verified in earlier stages of MS or RRMS. The median age for the control patients was 5 years lower than for the MS patients. The variation could introduce a higher degree of association between CHI3L1 and the degeneration of lipofuscin-positive neurons, given that elevated levels of neuronal lipofuscin are a common characteristic of ageing. However, the data from the CPZ model supports the appearance of CHI3L1  +  neurons being associated with inflammation-related neuronal stress in the brain. Our study discerned the association between lipofuscins and CHI3L1  +  neurons by comparing the CHI3L1 and Sudan Black staining in the same regions of identical tissue blocks. The study encountered a methodological constraint due to our inability to perform double staining of CHI3L1 and lipofuscin, resulting from the incompatibility between the immunostaining and chemical staining techniques applied to each. Despite an extensive literature search, we couldn’t find any verified protocols for performing double staining with Sudan Black-B and an antibody. Remarkably, Sudan Black B (Sigma-Aldrich®), a unique lipid staining method for Lipofuscin, enables the identification of senescent cells (Evangelou & Gorgoulis, 2017). Our study identified cortex neurons based on distinct morphological features. Colocalization analysis confirmed the astrocyte identity of CHI3L1  +  cells with an astrocyte morphology. The study also found that antigen-presenting cells with a microglia/macrophage morphology were CHI3L1 positive. The microglial/macrophage identity of these cells was confirmed by the colocalization of CHI3L1 with HLA-DR. Several previous studies by us and others have found that HLA-DR  +  cells in MS-lesions are macrophage/microglia (Bö et al., 1994). Future studies could focus on exploring colocalization patterns of CHI3L1  +  neurons using various neuronal markers.

In addition to the known role of CHI3L1 in neuroinflammation, this study highlights some previously unidentified roles of CHI3L1 in MS pathology, by identifying its association with neurodegeneration in GM and BBB abnormalities in the WM. These findings are further strengthened by the observed association of CHI3L1 positivity with the inflammation of the choroid plexus in the CPZ mouse (Supplementary Figure 2), since choroid plexus inflammation is correlated to neurodegeneration and malfunction of BBB (Čarna et al., 2023; Wolburg & Paulus, 2010).

## Conclusions

This research elucidates further roles of CHI3L1 in the pathogenesis of MS, particularly its progressive form. The central findings predominantly underscore CHI3L1's association with neuronal deterioration, pre-lesion pathology, and inflammatory changes in MS, thereby affirming its contribution to the disease's progression. While the study corroborates earlier findings linking CHI3L1 with macrophage infiltration and glial activation, it expands our understanding by demonstrating a higher level of CHI3L1 immunopositivity in the cortex of MS patients. Broadly, this investigation has revealed new aspects of CHI3L1's role in MS pathology. The study highlights the correlation of CHI3L1 with neuronal degradation in GM, abnormalities in the BBB, and the occurrence of CHI3L1 positivity tied to inflammation of the choroid plexus as observed in a CPZ mouse model.

## Supplemental Material

sj-docx-1-asn-10.1177_17590914231198980 - Supplemental material for An Association of Chitinase-3 Like-Protein-1 With Neuronal Deterioration in Multiple Sclerosis Click here for additional data file. Supplemental material, sj-docx-1-asn-10.1177_17590914231198980 for An Association of Chitinase-3 Like-Protein-1 With Neuronal Deterioration in Multiple Sclerosis by Intakhar Ahmad, Stig Wergeland, Eystein Oveland and Lars Bø in ASN Neuro
