# Supplementary material for: An Association of Chitinase-3 Like-Protein-1 With Neuronal Deterioration in Multiple Sclerosis
Source: ASN Neuro. 2023 Dec 7;15:17590914231198980. doi: 10.1177/17590914231198980 (PMC10710113; doi:10.1177/17590914231198980)

**Supplementary figure. 1: Density of CHI3L1+ cells in white matter tissue**

Both images are stained for CHI3L1 (brown) and myelin proteolipid protein, PLP (pink). **A)** Control white matter (WM) shows very few CHI3L1+ cells; positive says are marked with black arrowheads **B**) Higher density of CHI3L1+ cells is observed in MS-WM; positive says are marked with yellow arrowheads, and the lesion area is marked with a *****. Scale bar = 20 μm.

**
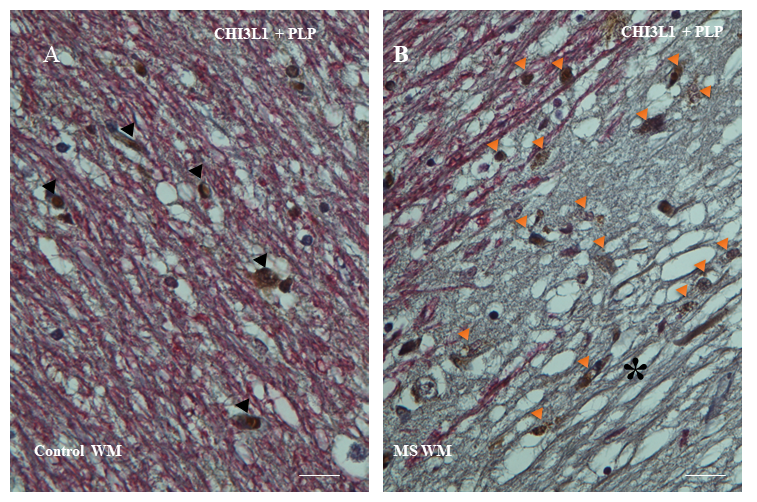
**

**Supplementary figure. 2: Inflammation of choroid plexus and its association with CHI3L1**

The images show DAB-stained (brown) slides from analogous regions in the mouse brain for two separate target molecules. **A)** Mac-3 immunopositivity indicates the level of inflammation in the choroid plexus and in the corpus callosum. **B**) Expression of CHI3L1 in the inflamed area in the brain matching the area in A. Scale bar = 20 μm.


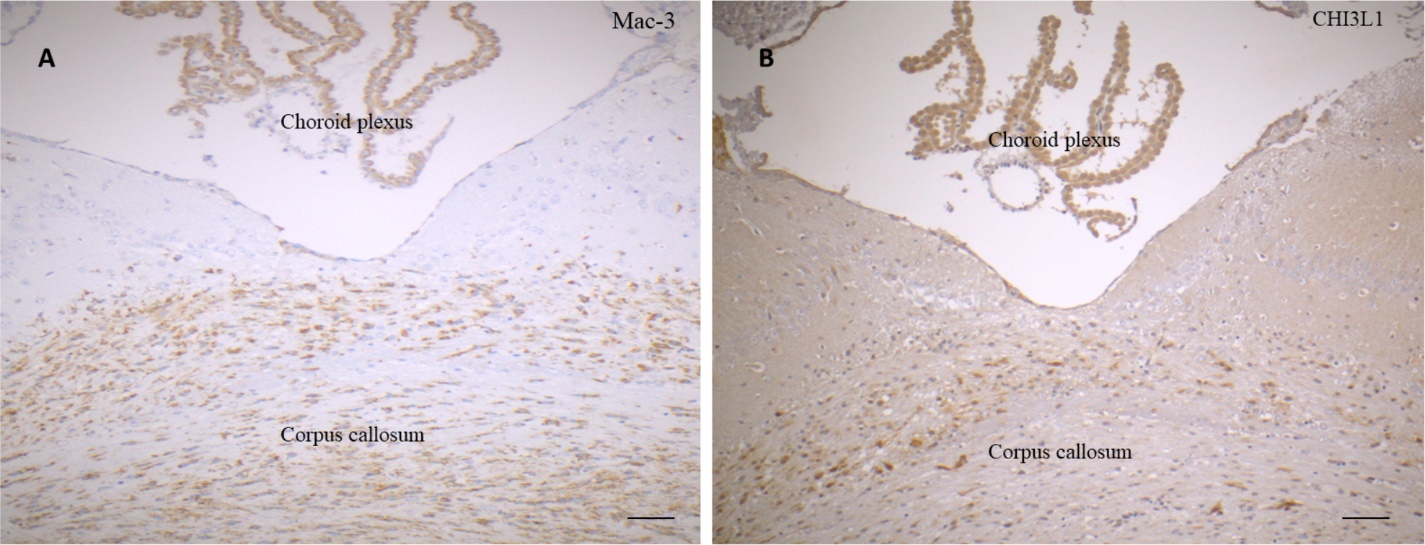

Supplement: sj-docx-1-asn-10.1177_17590914231198980 - Supplemental material for An Association of Chitinase-3 Like-Protein-1 With Neuronal Deterioration in Multiple Sclerosis [file sj-docx-1-asn-10.1177_17590914231198980.docx]
